# Man with Abdominal Bloating, Weight Loss

**DOI:** 10.24908/pocusj.v10i01.18109

**Published:** 2025-04-15

**Authors:** Sara Greenwald, Mario Ramos, Brian Kohen

**Affiliations:** 1 Department of Emergency Medicine, Memorial Hospital West, Pembroke Pines, FL, USA; 2 Department of Emergency Medicine, HCA Florida Kendall Hospital, Miami, FL, USA

**Keywords:** Intussusception, pseudo kidney sign, POCUS, target sign

## Abstract

Point of care ultrasound (POCUS) is useful in diagnosing intussusception. In this case file, we describe a 69-year-old man presenting with nausea, vomiting, left upper quadrant abdominal pain and recent weight loss who had findings concerning for intussusception on POCUS. This led the emergency provider to order computed tomography (CT) which confirmed the diagnosis.

## Presentation

**Figure 1.**
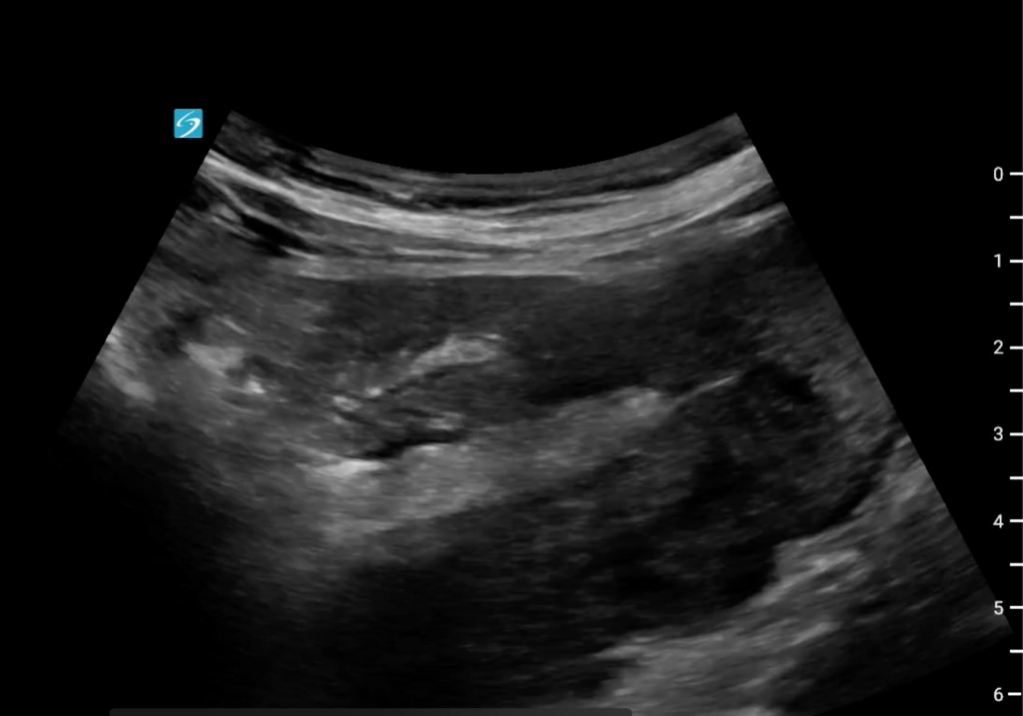
Pseudokidney sign – telescoping small bowel seen in long-axis resembling a kidney.

**Figure 2.**
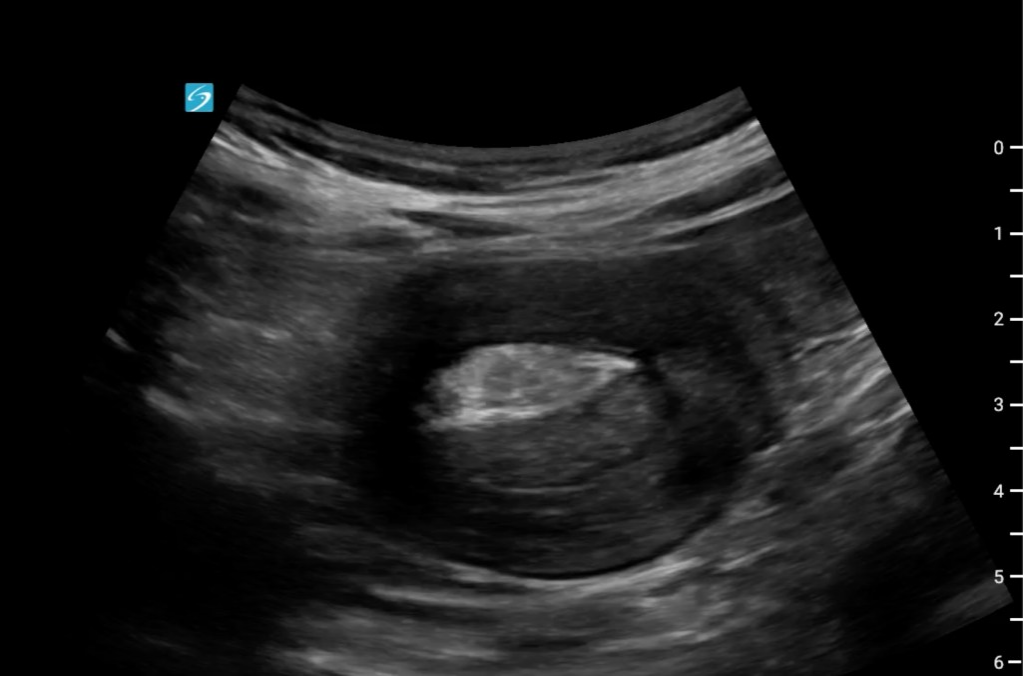
Target sign – telescoping small bowel seen in short-axis resembling a target.

A 69-year-old man presented to the emergency department with two days of persistent nausea and vomiting associated with generalized weakness, left upper quadrant discomfort and a recent forty-pound weight loss. He was admitted to the hospital two weeks prior for similar symptoms. At that point, he was found to have an acute kidney injury and was ultimately discharged. During his second visit, his vital signs were significant for a blood pressure of 91/79 but were otherwise within normal limits. On physical examination, the patient's abdomen was soft, non-tender, and non-distended. He appeared fatigued with dry oral mucous membranes. POCUS of his abdomen was performed which showed telescoping bowel in a longitudinal orientation, known as pseudokidney sign (Figure 1) and target sign (Figure 2) when viewed transversely. These findings were concerning for intussusception and prompted the provider to obtain CT of his abdomen and pelvis. CT confirmed a diagnosis of small bowel intussusception involving the proximal jejunum with an associated small bowel obstruction. The patient was admitted to the hospital where he underwent small bowel resection and evisceration of a jejunal mass, which was later found to be adenocarcinoma.

## Discussion

Intussusception is a rare diagnosis in adults, accounting for only 5% of all cases of intussusception [1]. In pediatric cases, ultrasound is 98-100% sensitive and 88-89% specific in diagnosing intussusception [1]. However, there is limited data about the use of POCUS to diagnose intussusception in adults. CT is currently considered the diagnostic test of choice for intussusception in adults, with a diagnostic accuracy ranging from 58-100% [2]. In adults, 65% of intussusception cases are caused by tumors, with structural abnormalities present in up to 90% of cases [1]. Signs consistent with intussusception on POCUS include pseudokidney and target sign, which are telescoping of the small bowel seen in long and transverse views, respectively [3].

However, more data is needed on POCUS test characteristics for intussusception in adults [3]. In our case, POCUS changed clinical management during a repeat hospital visit as it raised suspicion for intussusception, prompted obtaining further imaging, and ultimately expedited definitive care.


